# Effectiveness of the Mobile Emergency Medical Services (SAMU): use of interrupted time series

**DOI:** 10.11606/s1518-8787.2019053001396

**Published:** 2019-11-18

**Authors:** Cátia C. Martins Oliveira, Hillegonda Maria Dutih Novaes, Airlane Pereira Alencar, Itamar S. Santos, Maria Cecilia T. Damasceno, Heraldo Possolo de Souza

**Affiliations:** I, II Fundação Oswaldo Cruz. Instituto René Rachou. Coordenação da Agenda 2030. Belo Horizonte, MG, Brasil; II Universidade de São Paulo. Departamento de Medicina Preventiva. Programa de Pós-Graduação em Saúde Coletiva. São Paulo, SP, Brasil; III Universidade de São Paulo. Instituto de Matemática e Estatística. Departamento de Estatística. São Paulo, SP, Brasil; IV Universidade de São Paulo. Faculdade de Medicina. Departamento de Clínica Médica. São Paulo. São Paulo, SP, Brasil; V Faculdade de Medicina do ABC. Faculdade de Medicina. Departamento de Clínica Médica. São Paulo, SP, Brasil

**Keywords:** Mobile Health Units, Myocardial Infarction, Hospital Mortality, Efficacy-Effectiveness Evaluation of Interventions, Interrupted Time Series Analysis

## Abstract

**OBJECTIVE:**

To evaluate the performance of the Mobile Emergency Medical Services (SAMU) in the ABC Region, using myocardial infarction as tracer condition.

**METHODS:**

The analysis of interrupted time series was the approach chosen to test immediate and gradual effects of the intervention on the study population. The research comprised adjusted monthly time series of the hospital mortality rate by myocardial infarction in the period between 2000 and 2011. Data were extracted from the Mortality Information System (SIM), using segmented regression analysis to evaluate the level and trend of the intervention before and after its implementation. To strengthen the internal validity of the study, a control region was included.

**RESULTS:**

The analysis of interrupted time series showed a reduction of 0.04 deaths per 100,000 inhabitants in the mortality rate compared to the underlying trend since the implementation of the Emergency Medical Services (p = 0.0040; 95%CI: −0.0816 – −0.0162) and a reduction in the level of 2.89 deaths per 100,000 inhabitants (p = 0.0001; 95%CI: −4.3293 – −1.4623), both with statistical significance. Regarding the control region, Baixada Santista, the difference in the result trend between intervention outcome and post-intervention control of −0.0639 deaths per 100,000 inhabitants was statistically significant (p = 0.0031; 95%CI: −0.1060 – −0.0219). We cannot exclude confounders, but we limited their presence in the study by including control region series.

**CONCLUSIONS:**

Although the analysis of interrupted time series has limitations, this modeling can be useful for analyzing the performance of policies and programs. Even though the intervention studied is not a condition that in itself implies effectiveness, the latter would not be present without the former, which, integrated with other conditions, generates a positive result. SAMU is a strategy that must be expanded when formulating and consolidating policies focusing on emergency care.

## INTRODUCTION

Prehospital emergency care has been increasingly relevant in our society, because of the need to structure emergency care, ensuring a shorter response time and better regulation of care flows. Countries that organized their emergency care systems focusing on prehospital care, regardless of the model adopted, achieved good results in terms of survival, time, and cost of care, especially for traumas and chronic degenerative diseases^1 , 2^ .

In Brazil, mobile emergency prehospital care has been offered since 2003, by the Mobile Emergency Medical Services (SAMU). Inspired by the French model, but with features of the American model, its main objectives are to reduce the number of deaths, length of hospitalization, and sequelae resulting from the lack of timely care. It is operated by the emergency regulation demanded by the user, and the conduct can take place by telephone or by dispatch of basic or advanced life support teams, for the severe cases that require a more complex intervention^2 , 3^ .

Recent reviews have found evidence of the beneficial effect of prehospital emergency care on the prognosis of important problems, such as traumas and stroke, for which the response time to start treatment is greatly important^4 - 6^ . Some authors involved in this topic in Brazil emphasize that SAMU promotes, in addition to the prehospital start of treatment at the site of the event, the immediate removal of the patient to the tertiary center, when indicated by protocol based on the best evidence^7 , 8^ .

Myocardial infarction has been pointed out as a problem especially indicated for studies evaluating the performance of prehospital emergency care, given its magnitude, sensitivity to hospital medical technologies, and impact on mortality and hospital lethality. Early diagnosing myocardial infarction and carrying out prehospital care increase the patient’s chance of survival^9^ .

Rigorous methods to assess the outcomes of interventions within policies and programs are required to determine their effectiveness. One of the study designs recommended to evaluate the effect of an intervention is the interrupted time series analysis, a quasi-experimental method used when randomized studies are not feasible^10^ . In this design, a series of observations of the same outcome is performed at multiple points in time, before and after the intervention (“interruption”) is implemented. This method controls the baseline level and trend when estimating the expected changes resulting from the implementation of the program^11 , 12^ .

This study used interrupted time series to evaluate the effectiveness of the mobile emergency medical services on the hospital mortality rate by myocardial infarction in a region of Brazil.

## METHODS

This is an ecological study with interrupted time series analysis, considered one of the most effective quasi-experimental designs to evaluate the longitudinal effect of interventions^10 , 11^ . In this type of study, the time series of a result of interest, that is, the continuous sequence of observations of a given outcome repeatedly taken (usually at equal intervals) over time, is used to establish an underlying trend that is “interrupted” by an intervention at a known moment. The hypothetical scenario occurs considering that the intervention was not implemented and the trend remains unchanged, which is called “counterfactual” (“expected” trend in the absence of the intervention given the preexisting trend). The counterfactual scenario allows evaluating the effectiveness of the intervention by examining the path of the trend in the post-intervention period^10 , 13 , 14^ .

The research was developed in the ABC Region, located in the state of São Paulo, comprising seven municipalities and a population of about 2,719,580 inhabitants. SAMU started to be implemented in 2004, in the municipality of Santo André, and expanded to the others between 2004 and 2005. Since some cities at the time were not big enough to constitute a regulation center of SAMU, agreements were held for the regional installation. Therefore, the center of Mauá is a reference for Rio Grande da Serra and Ribeirão Pires, the one of Santo André is interconnected with São Caetano, and Diadema and São Bernardo have their own centers. SAMU covers all localities with basic and advanced life support ambulances, as well as motorcycle ambulances.

The rate of hospital mortality by myocardial infarction (MI) was evaluated based on the diagnosis of death with the codes I20 to I24 in the 10^th^ revision of the International Classification of Diseases (ICD-10). The monthly data were extracted from the *Sistema de Informações sobre Mortalidade* (SIM – Mortality Information System) of the *Departamento de Informática do Sistema Único de Saúde* (DATASUS – Informatics Department of the Brazilian Unified Health System), which captures about 90% of all deaths in the country, referring to public and private hospitals.

The numerator corresponded to the number of deaths by MI in the population aged 40 years or older living in the municipalities that compose the ABC Region. The denominator referred to the estimated population, according to the census of the Brazilian Institute of Geography and Statistics (IBGE), for the same age group, region, and period of the study. To allow comparison over time and between regions, the values were converted into rates per 100,000 inhabitants and standardized by the direct method.

To strengthen the internal validity of the study, a control region was included (group not exposed to the intervention), using the same outcome and observation period. This strategy is particularly valuable when there are other changes over time, unrelated to the intervention studied, but that can affect the results of the outcome^14 - 16^ .

For the selection of the control region, we analyzed the localities in the state of São Paulo whose municipalities had not implemented SAMU before 2011, according to a document provided by the Brazilian Ministry of Health. Then, to find comparable controls, those with p-value greater than 0.05 were identified for the covariates that measure the difference in level and trend during the pre-intervention period, included in the final model. The pre-intervention trend does not need to have exactly the same values as a randomized trial, but it must have a similar trend for the control group to be sustainable. Further methodological details can be found in Linden and Adams^17^ .

To compare the regions, the following aspects were selected: socioeconomic indicators (illiteracy rate of the population aged 15 years or older; average income, in R$; and total public health expenditure per inhabitant, in R$); and primary, secondary, and tertiary health care indicators (estimated population coverage of primary health care teams, family health doctors, or community physicians per 100,000 inhabitants; percentage of hospitalizations due to conditions sensitive to primary health care; total hospitalization beds of SUS per 1,000 inhabitants; total adult or coronary intensive care unit beds per 100,000 inhabitants; angioplasty rate standardized by sex and age per 100,000 inhabitants aged 20 years or older; percentage of the population covered by supplementary health care plans and insurance). The data sources for these indicators were the website of the project *Regiões e Redes* (Regions and Networks) and the website of the *Projeto Avaliação do Desempenho do Sistema de Saúde* (PROADESS – Project Health System Performance Assessment) of Fundação Oswaldo Cruz. For the comparative analysis of the regions, the years 2000 (start of the study) and 2010 (close to the study completion) were chosen. Because of the unavailability of socioeconomic and organizational regional information of health care in the public databases for 2011, we decided to use 2010 for comparison.

The study comprised monthly time series between 2000 and 2011, divided into three segments: pre-intervention period (January 2000 to December 2003), implementation phase of the intervention (January 2004 to December 2004), and post-intervention period (January 2005 to December 2011). It is worth noting that this study was completed in 2011 because of the inclusion of the control region in the analysis. As the largest expansion of prehospital emergency care occurred from 2012 onward, when the *Política Nacional de Atenção às Urgências* (National Emergency Care Policy) was reformulated, giving rise to the urgency and emergency network, it would not be possible to identify after that a control region similar to the intervention region for evaluating possible concurrent explanations.

The rate of hospital mortality by myocardial infarction was analyzed by adjusting the segmented regression model, including as covariates the time, the indicator variable equal to one after the intervention and zero before the intervention (and the interaction between these two variables), to evaluate the effects of change on level and trend in the period before and after the intervention. Seasonality was corrected, including month indicator variables (with January as reference)^1^ .

The graphs of residue and sample and partial autocorrelation function (ACF and partial ACF) were used to verify autocorrelation in the residue and properties of stationarity and normality, to select the most appropriate and statistically parsimonious models. The Ljung-Box test indicated that the model is appropriate to describe the linear dependence between successive repetitions^18^ .

## RESULTS

In the ABC Region, myocardial infarction was the main isolated cause of death in recent years. Between 2000 and 2011, 12,559 deaths of individuals aged 40 years or older were registered. Table 1 shows the monthly data of the standardized monthly hospital mortality rate for this period.

**Table 1:** Adjusted rate of monthly hospital mortality by myocardial infarction. ABC Region, SP, 2000-2011.

Months/Years	2000	2001	2002	2003	2004	2005	2006	2007	2008	2009	2010	2011
January	12.56	9.81	11.97	10.99	10.59	9.92	9.53	7.23	8.86	7.08	8.64	8.48
February	9.49	7.79	9.69	9.58	9.61	9.79	6.44	7.46	8.29	8.29	5.40	7.41
March	10.37	10.39	11.26	11.98	12.68	11.15	9.93	7.12	9.43	7.96	5.94	7.30
April	9.06	9.95	9.55	14.37	13.10	9.24	10.07	8.15	9.65	7.96	9.39	8.80
May	14.46	12.12	12.11	12.40	15.33	12.23	10.47	12.86	7.84	9.07	10.04	7.94
June	14.46	11.97	12.97	12.68	13.79	10.87	12.89	13.90	9.65	9.29	8.10	9.98
July	15.34	12.69	14.11	14.09	14.49	11.82	11.41	9.42	10.45	10.51	9.39	7.09
August	12.85	12.26	12.11	11.69	13.65	11.69	12.62	9.42	8.06	9.18	9.93	8.27
September	14.31	9.52	10.97	13.24	10.73	12.10	10.47	7.12	8.40	8.74	7.02	7.94
October	13.14	11.83	11.68	14.09	11.98	11.82	10.61	8.61	7.27	9.07	9.72	7.51
November	13.44	10.67	11.54	13.10	9.89	9.11	10.47	9.99	6.02	9.07	8.31	8.80
December	10.52	12.98	10.40	10.28	11.01	9.51	11.01	8.04	8.52	8.07	9.83	6.76

In this study, the statistical analysis empirically tested the hypothesis of significant reduction in the trend and level of the hospital mortality rate by MI in adults older than 40 years after the implementation of prehospital emergency care that took place in 2004. Since in a time series analysis the terms of error of consecutive observations can be correlated, the portions of the model residue were inspected as a function of time to ensure that no pattern suggesting autocorrelation was evident.

**Figure 1:**
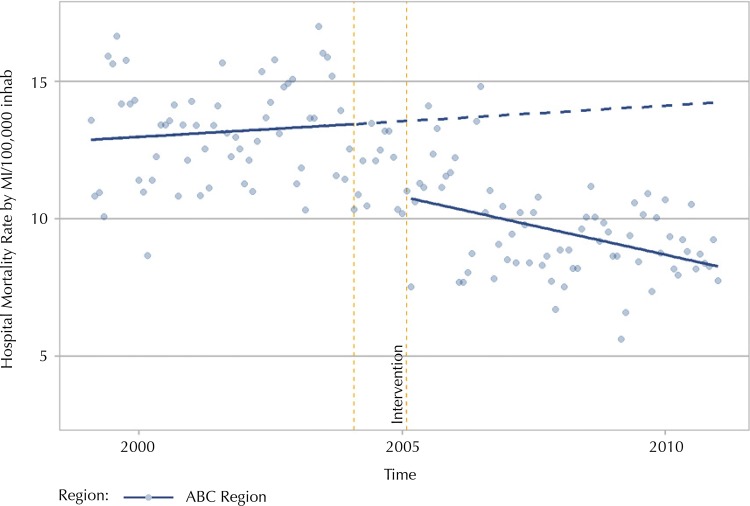
Estimated and predicted effect of the hospital mortality rate by myocardial infarction for the period before and after the mobile emergency medical services were implemented. ABC Region, SP, 2000-2011.

Figure 1 shows the graph of the outcome estimated and predicted effect, considering the period before and after the intervention was implemented in the ABC Region. The analysis of interrupted time series showed a reduction of 0.04 deaths per 100,000 inhabitants in the monthly rate of hospital mortality by MI compared to the underlying trend (p = 0.0040; 95%CI: 0.0816 – −0.0162) and a reduction in the level of 2.89 deaths per 100,000 inhabitants in the monthly rate of hospital mortality by MI (p = 0.0001; 95%CI: −4.3293 – −1.4623), with statistical significance. The findings indicate that, from 2005 onward, an immediate drop in the rate of hospital mortality by myocardial infarction and reduction of the trend took place, gradually remaining this way over the period of analysis.

To reduce the possibility of confounding, we chose to work with a control group comparable to the intervention group. The only region in the state of São Paulo that met the established criteria, that is, not having SAMU implemented during the analysis period and presenting covariates similar to those of the ABC Region, was the region of Baixada Santista.

Table 2 presents demographic and economic indicators, as well as primary, secondary, and tertiary care indicators, to compare both regions of analysis for the years of 2000 and 2010. Considering that the rate of angioplasty (standardized by sex and age per 100,000 inhabitants aged 20 years or older) presented increasing values in both regions between 2000 and 2010, we decided to include the annual trend of this indicator, as Table 3 shows.

**Table 2:** Socioeconomic and organizational indicators of Baixada Santista and ABC Region, SP, 2000 and 2010.

Analysis indicators	Control region		Region of intervention	
	
	Baixada Santista		ABC Region	
	
	2000	2010	2000	2010
Illiteracy rate of the population aged 15 years or older	5.7	4.0	4.9	3.2
Average income (in R$)	834.16	934.48	858.19	1026.94
Percentage of the population covered by supplementary health care plans and insurance	29.5	39.7	51.7	53.6
Estimated population coverage of primary health care teams	23.41	27.36	18.07	20.43
Family health doctors or community physicians per 100,000 inhabitants	11.9	32.6	15.7	35.9
Percentage of hospitalizations due to conditions sensitive to primary health care	6.3	5.9	6.3	6.9
Total number of hospitalization beds in SUS per 1,000 inhabitants	1.89	1.82	2.27	2.02
Adult/coronary ICU beds per 100,000 inhabitants	10.3	13.8	14.8	15.9
Standardized angioplasty rate by sex and age per 100,000 inhabitants aged 20 years or older	17.7	18.1	29.9	39.9
Total public health expenditure per inhabitant (in R$)	167.3	530.5	154.2	515.7

SUS: Brazilian Unified Health System; ICU: intensive care unit.

Source: Tabnet/Datasus and Project Health System Performance Assessment (PROADESS)

**Table 3:** Rate of angioplasty standardized by sex and age per 100,000 inhabitants aged 20 years or older. ABC Region and Baixada Santista, state of São Paulo, Southeast Region, 2000-2011.

Health Region	2000	2001	2002	2003	2004	2005	2006	2007	2008	2009	2010	2011
ABC Region	29.9	29.6	32.7	35.4	29.8	29.0	25.9	28.1	33.2	39.7	39.9	41.7
Baixada Santista	17.7	18.9	20.2	19.7	18.8	18.6	18.9	20.9	23.2	19.8	18.1	17.4
São Paulo, Brazil	31.6	38.4	40.2	41.5	38.1	37.2	37.4	36.6	45.7	46.8	50.8	52.1
Southeast	24.4	29.9	31.7	33.6	30.7	29.1	31.3	31.8	38.7	43.1	46.0	48.8
Brazil	21.8	27.0	29.4	30.6	29.1	28.5	30.9	31.1	38.9	41.9	44.2	46.7

Source: Project Health System Performance Assessment (PROADESS)

**Figure 2:**
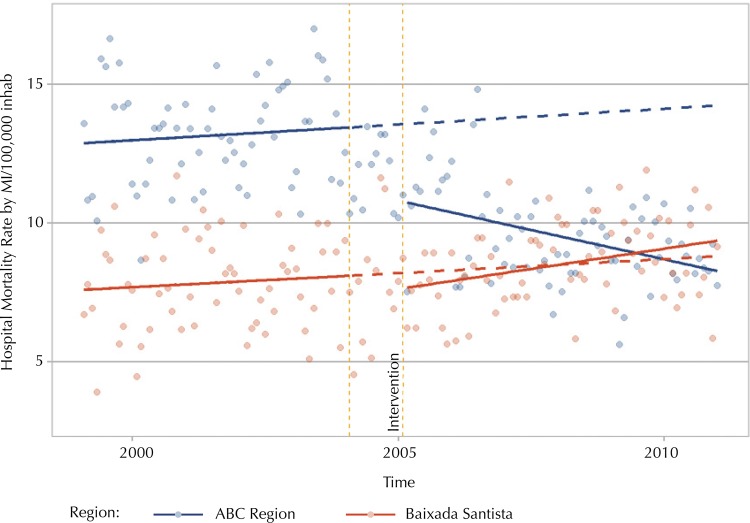
Estimated and predicted effect of the hospital mortality rate by myocardial infarction for the period before and after the mobile emergency medical services were implemented. ABC Region and Baixada Santista, SP, 2000-2011.

Figure 2 shows the change in level and trend of the hospital mortality due rate by myocardial infarction in individuals aged 40 years or older for the ABC Region and Baixada Santista, highlighting the control region as counterfactual. All results refer to changes after the adjustment for seasonal variation and autocorrelation. Compared to the control region, the ABC Region presented an ascending trend in the period before the implementation of SAMU, corresponding to 2.3 deaths per 100,000 inhabitants. The difference of −0.0639 in the trend of the post-intervention result between the intervention and control group was statistically significant (p = 0.0031; 95%CI: −0.1060 – −0.0219). A statistically significant change in level (degree) was not identified in the transition from the first to the second segment (p = 0.257), when comparing the two regions. These results suggest a change of trend between the two groups, which can strengthen the hypothesis of the study regarding the effect of the intervention implemented in the region from 2004.

## DISCUSSION

The design of interrupted time series involves estimating the effect of an intervention evaluating whether there is immediate impact (change in level) or progressive impact (change in trend) in the values of the series^12 , 13^ . In the ABC Region, the statistically significant change in the trend observed in the hospital mortality rate by myocardial infarction, mainly from 2004, suggests some hypotheses, among which a possible effect of the prehospital emergency care.

The *V Diretriz da Sociedade Brasileira de Cardiologia sobre Tratamento do Infarto Agudo do Miocárdio* (Fifth Guideline of the Brazilian Society of Cardiology on the Treatment of Myocardial Infarction) (2015)^19^ considers prehospital services as one of the main mechanisms for the reduction of deaths resulting from this disease, because they favor early diagnosis and ensure the referral of patients. However, there are few studies showing the effectiveness of this intervention in the country^20 , 21^ .

The reduction in the hospital mortality rate by MI after the introduction of prehospital emergency care is consistent with results from previous studies, which reported beneficial effects on the time of response^6 , 20 , 22^ . In the United States, even with the advances in the treatment of MI within hospitals, several studies highlight the importance of resuscitation and defibrillation in prehospital treatment, because they reduce the time elapsed between the onset of symptoms and the start of treatment^23^ .

In a study conducted in Rwanda, it was observed that a well-structured mobile prehospital care can lead to a gradual and sustained decrease in the mortality rate by the disease^24^ . A research conducted in Canada on mobile emergency units, with interrupted time series, suggested that mobile prehospital care can improve the quality of care in the cases of stroke and MI; however, clinically potential effects need to be better studied^6^ . Other studies, however, pointed out little or no effect of this modality of care on hospital mortality, even by MI, suggesting that it may have different effects in diverse situations and that its impact cannot be considered unquestionable^25^ .

Although in Brazil there are no researches with the interrupted time series method for SAMU, the effect of mobile prehospital care was identified in some publications. An ecological study with longitudinal design observed a beneficial effect of SAMU for mortality by stroke in men and mortality by MI in women in the elderly population of the state of Minas Gerais^20^ .

Results of a research conducted in the state of Rio de Janeiro, also with time series, showed that SAMU can reduce the mean length of hospital stay, especially for stroke, in both sexes, and trauma, for men^26^ . A study produced by the *Instituto de Pesquisa Econômica e Aplicada* (Ipea – Institute of Economic and Applied Research) stressed that the rate of hospital deaths by MI is higher in municipalities without units of SAMU, compared with those with them, although the difference is not expressive^7^ .

Even considering that SAMU represents a prehospital mobile emergency equipment, which can contribute to the health status in which patients reach hospitals, several other factors that improve patient survival must be considered when interpreting results. Thus, the implementation of preventive measures, focusing on risk factors related to MI, as well as advances in medical care, such as the use of beta-blockers and stent angioplasty, may substantially affect the rate of hospital admission and mortality^7 , 24^ .

The incorporation of a control group, using the same outcome in a group not exposed to the intervention, adds legitimacy when seeking to control possible biases, such as those mentioned above^16 , 17^ . The statistical analysis of time series, along with the analysis of threat to validity, may provide useful information about the effectiveness of the intervention^25^ . In this study, in the pre-intervention period, the difference in trend between the ABC Region and Baixada Santista was very close: 0.001. This suggests that, without intervention, the post-2004 trend in the ABC Region would probably have been similar to that of the Baixada Santista.

Considering that the different regions of the state are heterogeneous regarding socioeconomic and organizational characteristics, we sought regional variables that describe important aspects possibly related to the behavior of the hospital mortality rate by MI. In this context, we also selected indicators of provision of primary, secondary, and tertiary care in both regions of study^27^ .

Between 2000 and 2010, the average income improved in both regions, and the illiteracy rate decreased in both as well. The coverage of supplementary health care plans and insurance increased significantly in Baixada Santista, from 29.5% to 39.7%, and had a slight increase from 51.7% to 53.6% in the ABC Region.

The estimated population coverage of primary care teams and family doctors increased in both regions between 2000 and 2010. However, the percentage of hospitalization due to conditions sensitive to primary health care increased in the intervention region and had a slight decrease in the control region. This indicator is used as a measure of effectiveness of primary health care, assuming that a more satisfactory performance of this level of care could result in a decrease in the risk of hospitalization for a set of diseases that includes hypertension and diabetes, important risk factors for MI.

The availability of beds in the ABC Region was higher in 2000 and 2010 than in the Baixada Santista, but still insufficient to meet the demands of the population. The lack of beds in hospitals tends to worsen the articulation between hospital and prehospital care, revealing important inequalities in the access to care.

Still during this period, although the rate of hospitalization beds of SUS per 1,000 inhabitants decreased in both regions, the total of adult or coronary intensive care unit beds per 100,000 inhabitants increased, as well as the values of the angioplasty rate standardized by sex and age per 100,000 inhabitants aged 20 years or older, which reflects the availability of cardiological care in the regions.

To better monitor this scenario, we analyzed the annual behavior of the angioplasty rate standardized by sex and age per 100,000 inhabitants aged 20 years or older, considering the same period of study. Both regions presented an important increase in the rate of cardiac surgeries between 2001 and 2003, period before the implementation of SAMU in the intervention region. From 2004, the rate of angioplasty reduces, a pattern that remains up to 2007, when this rate starts to increase over the subsequent years. However, in Baixada Santista, a downward trend starts from 2009 on.

Considering in the analysis other interventions that may have been introduced in the same period of study, it is worth highlighting the implementation of the *Unidades de Pronto Atendimento* (UPA – Emergency Care Units), which have been important in the diagnosis of emergencies in the care of cardiovascular diseases, especially myocardial infarction, because they are intermediate units between primary health care and hospital emergencies. However, although formulated in 2008, its large-scale implementation in the country took place between 2011 and 2016, interval not included in this study^28^ .

Concerning the adoption of the anti-smoking law formulated in 2007, its implementation in fact occurred in the state of São Paulo in 2009 with Law no. 13,541^29^ . This measure emphasized the prohibition of the use of cigarettes in public and private environments, except residences and places of religious worship and intended for the consumption of tobacco products, aiming to reduce mortality from chronic diseases, with emphasis on cancer and cardiovascular disease^30^ .

The greatest strength of this study was, besides using the quasi-experimental design of time series with control group, controlling differences in both the pre-existing level and trend. However, one of the limitations of our study is its low power of generalization; therefore, its conclusions should only be applied to populations with a similar profile. Conflicting results may be part of the differences in the local context. Furthermore, one must consider the role of SAMU within the other components of the emergency network in the different territories that compose the ABC Region. In this sense, in community-based studies, the use of a quasi-experimental design will often request both quantitative and qualitative approaches to reach a broader conclusion about the effects of the intervention under study^27 ,, 30 , 31^ .

Finally, the unexpressive result (although statistically significant) in the post-intervention trend of the hospital mortality rate by MI in the ABC Region may lead to an interpretation that the effect was of low impact. However, in the case of ecological studies, the effects occur equally in the population as a whole. Thus, findings with minor effects are expected, and this does not mean a low real effect on the studied phenomenon. In addition, the studied intervention may be a condition that in itself does not imply effectiveness, but without which this same effectiveness would not be present, integrating a set of conditions that, put together, generate a positive result.

## CONCLUSION

The complexity of seeking to establish the effect of a given intervention lies in the fact that almost all observed phenomena have multiple causes. From this perspective, the proposal of quasi-experimental designs by tracer conditions or marker events has been a recurring practice, since it is difficult, in many situations, to isolate the causes and effects of the phenomena of interest.

This study reinforces the usefulness of the quasi-experimental approach of interrupted series, making it a viable option for intervention analysis in health policies when randomized studies are not feasible. The results suggest that, although the effectiveness of SAMU cannot be considered as acquired, it is a strategy whose expansion needs to considered in the formulation and consolidation of policies focusing on emergency care.
