# Supplementary material for: Effectiveness of the Mobile Emergency Medical Services (SAMU): use of interrupted time series
Source: Rev Saude Publica. 2019 Nov 18;53:99. doi: 10.11606/s1518-8787.2019053001396 (PMC6863106; doi:10.11606/s1518-8787.2019053001396)
Supplement: Translation

# Translation: Efetividade do serviço móvel de urgência (Samu): uso de séries temporais interrompidas

**Authors:** Cátia C. Martins Oliveira, Hillegonda Maria Dutih Novaes, Airlane Pereira Alencar, Itamar S. Santos, Maria Cecilia T. Damasceno, Heraldo Possolo de Souza

**Affiliations:** I Fundação Oswaldo Cruz. Instituto René Rachou. Coordenação da Agenda 2030. Belo Horizonte, MG, Brasil; II Universidade de São Paulo. Departamento de Medicina Preventiva. Programa de Pós-Graduação em Saúde Coletiva. São Paulo, SP, Brasil; III Universidade de São Paulo. Instituto de Matemática e Estatística. Departamento de Estatística. São Paulo, SP, Brasil; IV Universidade de São Paulo. Faculdade de Medicina. Departamento de Clínica Médica. São Paulo. São Paulo, SP, Brasil; V Faculdade de Medicina do ABC. Faculdade de Medicina. Departamento de Clínica Médica. São Paulo, SP, Brasil

**Keywords:** Unidades Móveis de Saúde, Infarto do Miocárdio, Mortalidade Hospitalar, Avaliação de Eficácia-Efetividade de Intervenções, Análise de Séries Temporais Interrompida

## Abstract

**OBJETIVO:**

Avaliar o desempenho do serviço de atendimento móvel de urgência (Samu) na região do Grande ABC, utilizando como condição traçadora o infarto agudo do miocárdio.

**MÉTODOS:**

A análise de séries temporais interrompidas foi a abordagem de escolha para testar efeitos imediatos e graduais da intervenção na população de estudo. A pesquisa compreendeu séries temporais mensais ajustadas da taxa de mortalidade hospitalar por infarto agudo do miocárdio no período entre 2000 e 2011. Os dados foram extraídos do Sistema de Informações sobre Mortalidade, usando a análise de regressão segmentada para avaliar o nível e tendência da intervenção antes e após sua implementação. Para fortalecer a validade interna do estudo, foi incluída uma região controle.

**RESULTADOS:**

A análise de séries temporais interrompidas mostrou redução de 0,04 mortes por 100.000 habitantes na taxa de mortalidade em relação à tendência subjacente desde a implantação do serviço de atendimento médico de urgência (p = 0,0040; IC95% -0,0816 – -0,0162) e uma redução no nível de 2,89 mortes por 100.000 habitantes (p = 0,0001; IC95% -4,3293 – -1,4623), ambos com significância estatística. Em relação à região controle, a Baixada Santista, a diferença da tendência do resultado entre desfecho de intervenção e controle pós-intervenção de -0,0639 mortes por 100.000 habitantes mostrou-se estatisticamente significativa (p = 0,0031; IC95% -0,1060 – -0,0219). Não podemos excluir confundimentos, mas limitamos sua presença no estudo incluindo séries de região controle.

**CONCLUSÕES:**

Embora a análise de séries temporais interrompidas tenha limitações, essa modelagem pode ser útil para a análise de desempenho de políticas e programas. Apesar de a intervenção estudada não ser uma condição que por si só implica na efetividade, a efetividade não estaria presente sem essa intervenção, que, integrada a outras condições, gera um resultado positivo. O Samu é uma estratégia cuja expansão precisa ser levada em consideração ao formular e consolidar políticas com foco nas urgências e emergências.

## INTRODUÇÃO

A atenção pré-hospitalar móvel vem ganhando cada vez mais relevância em nossa sociedade, em virtude da necessidade de estruturar o atendimento à urgência, assegurando menor tempo de resposta e melhor regulação dos fluxos de atenção. Países que organizaram seus sistemas de atendimento às urgências com foco na atenção pré-hospitalar, independentemente do modelo adotado, alcançaram bons resultados em termos de sobrevida, tempo e custo de assistência, especialmente no caso de traumas e doenças crônico-degenerativas^1 , 2^ .

No Brasil a atenção pré-hospitalar móvel de urgência é ofertada desde 2003, por meio do serviço de atendimento móvel de urgência (Samu). Inspirado no modelo francês, mas com atributos do modelo americano, tem como principais objetivos a redução do número de óbitos, do tempo de internação e das sequelas decorrentes da falta de socorro oportuno. É operado por meio da regulação de urgência demandada pelo usuário, e a conduta pode ser telefônica ou por despacho de equipes de suporte básico ou avançado de vida, para os casos graves que necessitam de intervenção mais complexa^2 , 3^ .

Recentes revisões encontraram evidências do efeito benéfico da atenção pré-hospitalar de urgência sobre o prognóstico de importantes agravos como traumas e acidente vascular cerebral, para os quais o tempo resposta para o início do tratamento é de suma importância^4^ . Alguns autores envolvidos com o tema no Brasil ressaltam que o Samu promove, além do início pré-hospitalar do tratamento no local de ocorrência do evento, a imediata remoção do paciente para o centro terciário, quando indicado por protocolo baseado nas melhores evidências^7 , 8^ .

O infarto agudo do miocárdio tem sido apontado como um agravo especialmente indicado para estudos de avaliação do desempenho da assistência pré-hospitalar de urgência, dada a sua magnitude, sensibilidade a tecnologias médicas hospitalares e impacto na mortalidade e na letalidade hospitalar. Diagnosticar precocemente o infarto agudo do miocárdio e iniciar os cuidados emergenciais aumenta a chance de sobrevida do paciente^9^ .

Métodos rigorosos para avaliar resultados das intervenções no âmbito das políticas e programas são necessários para determinar sua efetividade. Entre os desenhos de estudo recomendados para avaliar o efeito de uma intervenção, há a análise de série temporal interrompida, método quase experimental, utilizado quando estudos randomizados não são viáveis^10^ . Nesse desenho, uma série de observações do mesmo desfecho é realizada em múltiplos pontos no tempo, antes e depois de implantada a intervenção (a “interrupção”). Esse método controla o nível e a tendência da linha de base ao estimar as mudanças esperadas resultantes da implantação do programa^11 , 12^ .

Este estudo utilizou séries temporais interrompidas para avaliar a efetividade do serviço de atendimento móvel de urgência sobre a taxa de mortalidade hospitalar por infarto agudo do miocárdio em uma região do Brasil.

## MÉTODOS

Estudo ecológico com análise de séries temporais interrompidas, considerado um dos mais efetivos desenhos quase experimentais para avaliar o efeito longitudinal de intervenções^10 , 11^ . Nesse tipo de estudo, a série temporal de um resultado de interesse, ou seja, sequência contínua de observações de um dado desfecho tomadas repetidamente (normalmente em intervalos iguais) ao longo do tempo, é usada para estabelecer uma tendência subjacente que é “interrompida” por uma intervenção em um momento conhecido. O cenário hipotético ocorre considerando que a intervenção não foi implantada e a tendência continua inalterada, o que se denomina como “contrafactual” (tendência “esperada” na ausência da intervenção dada a tendência pré-existente). O cenário contrafactual permite avaliar a efetividade da intervenção examinando a – trajetória da tendência no período pós-intervenção^10 , 13 , 14^ .

A pesquisa foi desenvolvida na região do Grande ABC, localizada no estado de São Paulo, composta por sete municípios e com uma população de aproximadamente 2.719.580 habitantes. O Samu começou a ser implantado em 2004, no município de Santo André, e expandiu para os demais entre os anos de 2004 e 2005. Como alguns na época não tinham porte para constituir uma central de regulação do Samu, foram realizados pactos para a instalação regional. Por isso, a base de Mauá é referência para Rio Grande da Serra e Ribeirão Pires, a de Santo André é interligada com São Caetano, e Diadema e São Bernardo possuem bases próprias. O Samu cobre todas as localidades com ambulâncias de suporte básico e avançado de vida, além de motolância.

Foi avaliada a taxa de mortalidade hospitalar por infarto agudo do miocárdio (IAM), com base no diagnóstico de morte com os códigos I20 a I24 na 10ª revisão da Classificação Internacional de Doenças (CID-10). Os dados mensais foram extraídos do Sistema de Informações sobre Mortalidade (SIM) do Departamento de Informática do Sistema Único de Saúde (Datasus), que capta cerca de 90% de todas as mortes ocorridas no país, referentes a hospitais públicos e privados.

O numerador correspondeu ao número de óbitos por IAM na população com 40 anos ou mais residente nos municípios que compõem a região do Grande ABC. O denominador se referiu à população estimada, segundo censo do Instituto Brasileiro de Geografia e Estatística (IBGE), para a mesma faixa etária, região e período do estudo. Para permitir a comparação ao longo do tempo e entre regiões, os valores foram convertidos em taxas por 100.000 habitantes e padronizados pelo método direto.

Para fortalecer a validade interna do estudo, foi incluída uma região controle (grupo não exposto à intervenção), utilizando o mesmo desfecho e período de observação. Essa estratégia é particularmente valiosa quando existem outras mudanças ao longo do tempo, não relacionadas à intervenção estudada, mas que podem influenciar os resultados do desfecho^14^ .

Para a seleção da região controle primeiro foram analisadas as localidades no estado de São Paulo cujos municípios não haviam habilitado o Samu antes de 2011, de acordo com documento fornecido pelo Ministério da Saúde. Em sequência, para encontrar controles comparáveis, foram identificados os que tinham p-valor maior que 0.05 para as covariáveis que mensuram a diferença de nível e tendência durante o período pré-intervenção, incluídos no modelo final. A tendência pré-intervenção não precisa ter exatamente os mesmos valores como em um ensaio randomizado, porém é importante que haja uma tendência similar para que haja sustentabilidade do grupo controle. Maiores detalhes metodológicos podem ser encontrados em Linden e Adams^17^ .

Foram elegíveis para comparar as regiões também os indicadores socioeconômicos (taxa de analfabetismo da população com 15 anos ou mais de idade;renda média *per capita,* em reais; e gasto público total em saúde por habitante, em R$) e marcadores da atenção básica, da média e da alta complexidade (cobertura populacional estimada de equipes de atenção básica, médicos de saúde da família ou médicos comunitários por 100.000 habitantes; percentual de internações por condições sensíveis à atenção primária; total de leitos de internação do Sistema Único de Saúde por 1.000 habitantes; total de leitos de unidade de terapia intensiva adulto ou coronariana por 100.000 habitantes; taxa de angioplastia padronizada por sexo e idade por 100.000 habitantes com 20 anos ou mais; percentual da população coberta por planos e seguros de assistência suplementar à saúde). As fontes de dados para esses indicadores foram o portal do projeto Regiões e Redes e o site do Projeto Avaliação do Desempenho do Sistema de Saúde (Proadess) da Fundação Oswaldo Cruz. Para análise comparativa das regiões, foram considerados os anos de 2000 (início do estudo) e 2010 (próximo à finalização do estudo). Devido à indisponibilidade de informações regionais de âmbito socioeconômico e organizacional da atenção à saúde nos bancos de dados públicos para o ano de 2011, optou-se por utilizar para comparação o ano de 2010.

O estudo compreendeu séries temporais mensais entre os anos 2000 e 2011, divididos em três segmentos: um período anterior (janeiro de 2000 a dezembro de 2003), a fase de implementação da intervenção (janeiro de 2004 a dezembro de 2004) e o período posterior à intervenção (de janeiro de 2005 a dezembro de 2011). Cabe ressaltar que este estudo foi finalizado em 2011 por causa da inclusão da região controle na análise. Como a maior expansão da atenção móvel pré-hospitalar de urgência ocorreu a partir de 2012, quando foi reformulada a Política Nacional de Atenção às Urgências, dando origem à rede de urgência e emergência, não seria possível depois disso identificar uma região controle similar à região de intervenção para avaliar possíveis explicações rivais.

A taxa de mortalidade hospitalar por infarto agudo do miocárdio foi analisada por meio do ajuste do modelo de regressão segmentada, incluindo como covariáveis o tempo, a variável indicadora igual a um após a intervenção e zero antes da intervenção e a interação entre essas duas variáveis, para avaliar os efeitos de mudança no nível e tendência no período antes e após a intervenção. A sazonalidade foi corrigida, incluindo variáveis indicadoras de mês (tendo janeiro como referência)^1^ .

Os gráficos de resíduos e da função de autocorrelação amostral e parcial (FAC e FAC parcial) foram utilizados para verificar autocorrelação nos resíduos e propriedades de estacionariedade e normalidade, a fim de selecionar os modelos mais adequados e parcimoniosos estatisticamente. O teste de Ljung-Box indicou que o modelo é apropriado para descrever a dependência linear entre os sucessivos retornos^18^ .

## RESULTADOS

Na região do Grande ABC, o infarto agudo do miocárdio foi a principal causa isolada de morte nos últimos anos. No período entre 2000 e 2011, ocorreram 12.559 óbitos em indivíduos com 40 anos ou mais. Os dados mensais da taxa de mortalidade hospitalar mensal padronizada para esse período estão apresentados na Tabela 1 .

**Tabela 1:** Taxa ajustada de mortalidade hospitalar mensal por infarto agudo do miocárdio. Região do Grande ABC, SP, 2000–2011.

Meses/Anos	2000	2001	2002	2003	2004	2005	2006	2007	2008	2009	2010	2011
Janeiro	12,56	9,81	11,97	10,99	10,59	9,92	9,53	7,23	8,86	7,08	8,64	8,48
Fevereiro	9,49	7,79	9,69	9,58	9,61	9,79	6,44	7,46	8,29	8,29	5,40	7,41
Março	10,37	10,39	11,26	11,98	12,68	11,15	9,93	7,12	9,43	7,96	5,94	7,30
Abril	9,06	9,95	9,55	14,37	13,10	9,24	10,07	8,15	9,65	7,96	9,39	8,80
Maio	14,46	12,12	12,11	12,40	15,33	12,23	10,47	12,86	7,84	9,07	10,04	7,94
Junho	14,46	11,97	12,97	12,68	13,79	10,87	12,89	13,90	9,65	9,29	8,10	9,98
Julho	15,34	12,69	14,11	14,09	14,49	11,82	11,41	9,42	10,45	10,51	9,39	7,09
Agosto	12,85	12,26	12,11	11,69	13,65	11,69	12,62	9,42	8,06	9,18	9,93	8,27
Setembro	14,31	9,52	10,97	13,24	10,73	12,10	10,47	7,12	8,40	8,74	7,02	7,94
Outubro	13,14	11,83	11,68	14,09	11,98	11,82	10,61	8,61	7,27	9,07	9,72	7,51
Novembro	13,44	10,67	11,54	13,10	9,89	9,11	10,47	9,99	6,02	9,07	8,31	8,80
Dezembro	10,52	12,98	10,40	10,28	11,01	9,51	11,01	8,04	8,52	8,07	9,83	6,76

Neste estudo, a análise estatística empiricamente testou a hipótese de redução significativa na tendência e no nível da taxa de mortalidade hospitalar por IAM em adultos com idade acima de 40 anos após a implementação da atenção pré-hospitalar móvel ocorrida em 2004. Como em uma análise de série temporal os termos de erro de observações consecutivas podem ser correlacionados, foram inspecionadas as parcelas dos resíduos do modelo em função do tempo para garantir que nenhum padrão sugerindo autocorrelação fosse evidente.

A Figura 1 apresenta o gráfico de efeito estimado e predito do desfecho, considerando o período antes e depois de implementada a intervenção na região do Grande ABC. A análise de séries temporais interrompidas mostrou redução de 0,04 mortes por 100.000 habitantes na taxa mensal de mortalidade hospitalar por IAM em relação à tendência subjacente (p = 0,0040; IC95%: -0,0816 – -0,0162) e uma redução no nível de 2,89 mortes por 100.000 habitantes na taxa mensal de mortalidade hospitalar por IAM (p = 0,0001; IC95%: -4,3293 – -1,4623), com significância estatística. Os achados indicam que, a partir de 2005, houve queda imediata na taxa de mortalidade hospitalar atribuída ao infarto agudo do miocárdio e redução da tendência, que se mantém de forma gradual ao longo do período de análise.

**Figura 1:** Efeito estimado e predito da taxa de mortalidade hospitalar por infarto agudo do miocárdio para o período antes e depois de implantado o serviço de atendimento móvel de urgência. Região do Grande ABC, SP, 2000–2011.

Fonte dos dados: Tabnet/DataSus

Para reduzir a possibilidade de confundimento, optou-se por trabalhar com um grupo controle comparável ao grupo de intervenção. A única região do estado de São Paulo que atendeu aos critérios estabelecidos, ou seja, não ter o Samu implementado durante o período de análise e apresentar valores de covariáveis semelhantes aos da região do Grande ABC, foi a região da Baixada Santista.

A Tabela 2 apresenta indicadores demográficos e econômicos, além de marcadores para atenção básica, média e alta complexidade, no intuito de comparar ambas as regiões de análise quanto aos anos de 2000 e 2010. Considerando que a taxa de angioplastia, padronizada por sexo e idade por 100.000 habitantes com 20 anos ou mais, apresentou entre os anos de 2000 e 2010 valores crescentes em ambas as regiões, optou-se por incluir a tendência anual desse indicador, conforme demonstrado na Tabela 3 .

**Tabela 2:** Indicadores socioeconômicos e organizacionais das regiões da Baixada Santista e Grande ABC, SP, 2000 e 2010.

Indicadores de análise	Região controle		Região de intervenção	
	
	Baixada Santista		Grande ABC	
	
	2000	2010	2000	2010
Taxa de analfabetismo da população com 15 anos ou mais de idade	5,7	4,0	4,9	3,2
Renda média *per capita* (em R$)	834,16	934,48	858,19	1026,94
Percentual da população coberta por planos e seguros de assistência suplementar à saúde	29,5	39,7	51,7	53,6
Cobertura populacional estimada das equipes de atenção básica	23,41	27,36	18,07	20,43
Médicos de saúde da família ou médicos comunitários por 100 mil habitantes	11,9	32,6	15,7	35,9
Percentual de internações por condições sensíveis à atenção primária	6,3	5,9	6,3	6,9
Total de leitos de internação do SUS por mil habitantes	1,89	1,82	2,27	2,02
Leitos de UTI adulto/coronariana por 100 mil habitantes	10,3	13,8	14,8	15,9
Taxa de angioplastia padronizada por sexo e idade por 100 mil habitantes com 20 anos ou mais	17,7	18,1	29,9	39,9
Gasto público total em saúde por habitante (em R$)	167,3	530,5	154,2	515,7

SUS: Sistema Único de Saúde; UTI: unidade de terapia intensiva.

Fonte dos dados: Tabnet/Datasus e Projeto Avaliação do Desempenho do Sistema de Saúde (Proadess)

**Tabela 3:** Taxa de angioplastia padronizada por sexo e idade por 100 mil habitantes com 20 anos ou mais. Regiões do Grande ABC e Baixada Santista, estado de São Paulo e Região Sudeste, 2000–2011.

Região de saúde	2000	2001	2002	2003	2004	2005	2006	2007	2008	2009	2010	2011
Grande ABC	29,9	29,6	32,7	35,4	29,8	29,0	25,9	28,1	33,2	39,7	39,9	41,7
Baixada Santista	17,7	18,9	20,2	19,7	18,8	18,6	18,9	20,9	23,2	19,8	18,1	17,4
São Paulo	31,6	38,4	40,2	41,5	38,1	37,2	37,4	36,6	45,7	46,8	50,8	52,1
Sudeste	24,4	29,9	31,7	33,6	30,7	29,1	31,3	31,8	38,7	43,1	46,0	48,8
Brasil	21,8	27,0	29,4	30,6	29,1	28,5	30,9	31,1	38,9	41,9	44,2	46,7

Fonte dos dados: Projeto Avaliação do Desempenho do Sistema de Saúde (Proadess)

A visualização de mudança no nível e tendência da taxa de mortalidade hospitalar por infarto agudo do miocárdio entre indivíduos com 40 anos ou mais para as regiões do Grande ABC e Baixada Santista está apresentada na Figura 2 , destacando a região controle como contrafactual. Todos os resultados referem-se a mudanças após o ajuste para a variação sazonal e autocorrelação. Na região do Grande ABC, quando comparada com a região controle, a tendência no período anterior à implantação do Samu era ascendente, correspondendo a 2,3 óbitos por 100.000 habitantes. A diferença de -0,0639 na tendência do resultado pós-intervenção entre grupo de intervenção e controle mostrou-se estatisticamente significativa (p = 0,0031; IC95% -0,1060 – -0,0219). Não foi identificada mudança de nível (degrau) estatisticamente significativa na transição do primeiro para o segundo segmento (p = 0,257) quando comparada as duas regiões. Esses resultados sugerem mudança de tendência entre os dois grupos, o que pode fortalecer a hipótese do estudo em termos de efeito da intervenção implementada na região a partir de 2004.

**Figura 2:** Efeito estimado e predito da taxa de mortalidade hospitalar por infarto agudo do miocárdio para o período antes e depois de implantado o serviço de atendimento móvel de urgência. Grande ABC e Baixada Santista, SP, 2000–2011.

Fonte dos dados: Tabnet/DataSus

## DISCUSSÃO

O desenho de séries temporárias interrompidas envolve estimar o efeito de uma intervenção avaliando se há impacto imediato (mudança de nível) ou impacto progressivo (mudança de tendência) nos valores da série^12 , 13^ . Na região do Grande ABC, a mudança de tendência estatisticamente significativa observada na taxa de mortalidade hospitalar por infarto agudo do miocárdio, principalmente a partir de 2004, sugere algumas hipóteses, entre elas um possível efeito da atenção pré-hospitalar móvel de urgência.

A V Diretriz da Sociedade Brasileira de Cardiologia sobre Tratamento do Infarto Agudo do Miocárdio (2015)^19^ considera como um dos principais mecanismos para a redução dos óbitos advindos dessa enfermidade os serviços pré-hospitalares, pois favorecem o diagnóstico precoce e garantem a referência de pacientes. No entanto, existem poucos estudos que demonstrem efetividade dessa intervenção no país^20 , 21^ .

A redução na taxa de mortalidade hospitalar por IAM após a introdução da atenção pré-hospitalar móvel de urgência é consistente com resultados de estudos anteriores, que relataram efeitos benéficos dela sobre o tempo de resposta^6 , 20 , 22^ . Nos Estados Unidos, mesmo com os avanços ocorridos no tratamento do IAM dentro dos hospitais, vários estudos destacam a importância da habilidade de ressuscitação e da desfibrilação no avanço do tratamento pré-hospitalar, por reduzirem o tempo decorrido entre o início dos sintomas e a instituição do tratamento^23^ .

Em estudo conduzido em Ruanda, foi observado que uma atenção pré-hospitalar móvel bem estruturada pode levar a um decréscimo gradual e sustentado na taxa de mortalidade pelo agravo assistido^24^ . Pesquisa realizada no Canadá sobre unidades móveis de urgência, com séries temporais interrompidas, sugeriu que a atenção pré-hospitalar móvel pode melhorar a qualidade do cuidado nos casos de AVC e IAM; entretanto, efeitos clinicamente potenciais precisam ser melhor estudados^6^ . Outros trabalhos, no entanto, apontaram pouco ou nenhum efeito dessa modalidade de atenção sobre a mortalidade hospitalar, inclusive por IAM, o que sugere que ela pode ter efeitos diferentes em diferentes situações e que seu impacto não pode ser considerado como indiscutível^25^ .

Embora no Brasil não haja pesquisas com o método de séries temporais interrompidas para o Samu, o efeito da atenção pré-hospitalar móvel foi identificado em algumas publicações. Um estudo de desenho ecológico, com delineamento longitudinal, observou um efeito benéfico da presença do Samu para a mortalidade por AVC no sexo masculino e mortalidade por IAM no sexo feminino em população idosa do estado de Minas Gerais^20^ .

Resultados de uma pesquisa realizada no estado do Rio de Janeiro, também com séries temporais, mostraram que o Samu pode reduzir o tempo médio de permanência hospitalar, principalmente para acidente vascular cerebral, em ambos os sexos, e trauma, para os homens^26^ . Um estudo produzido pelo Instituto de Pesquisa Econômica e Aplicada (Ipea) destacou que a taxa de óbitos hospitalares por IAM é maior nos municípios que não possuem unidades do Samu, comparados com aqueles que possuem, embora a diferença não seja expressiva^7^ .

Mesmo considerando que o Samu representa um equipamento móvel pré-hospitalar de urgência, que pode contribuir para o estado de saúde em que os pacientes chegam aos hospitais, diversos outros fatores que melhoram a sobrevida do paciente devem ser considerados na interpretação dos resultados. Assim, a implantação de medidas preventivas, com foco nos fatores de risco relacionados ao IAM, bem como avanços no cuidado médico, como o uso de betabloqueadores e a angioplastia com *stent* , podem interferir substancialmente na taxa de admissão e mortalidade hospitalar^7 , 24^ .

A incorporação de um grupo controle, utilizando o mesmo desfecho em um grupo não exposto à intervenção, adiciona legitimidade ao procurar controlar possíveis vieses, como os citados acima^16 , 17^ . A análise estatística de séries temporais, junto com a análise de ameaça à validade, pode prover informações úteis sobre a efetividade da intervenção^25^ . Neste estudo, no período pré-intervenção, a diferença de tendência entre as regiões do Grande ABC e Baixada Santista foi bastante próxima, de 0,001. Isso sugere que, sem intervenção, a tendência pós-2004 no Grande ABC provavelmente teria sido semelhante à da Baixada Santista.

Considerando que as diferentes regiões do estado são heterogêneas em relação às características socioeconômicas e organizacionais, buscou-se trazer variáveis regionais que descrevessem importantes aspectos possivelmente relacionados ao comportamento da taxa de mortalidade hospitalar por IAM. Nesse contexto, foram selecionados também marcadores de oferta de serviços da atenção básica, média e alta complexidade em ambas as regiões de estudo^27^ .

Entre os anos de 2000 e 2010, a renda média *per capita* melhorou em ambas as regiões, e a taxa de analfabetismo apresentou queda tanto no Grande ABC quanto na Baixada Santista. A cobertura de planos e seguros de assistência suplementar à saúde aumentou de forma expressiva na Baixada Santista, de 29,5% para 39,7%, e no Grande ABC observou-se um ligeiro aumento de 51,7% para 53,6%.

Houve crescimento da cobertura populacional estimada de equipes da atenção básica e médicos da família entre os anos de 2000 e 2010 em ambas as regiões. No entanto, o percentual de internação por condições sensíveis à atenção primária aumentou na região de intervenção e teve uma ligeira redução na região controle. Esse indicador é utilizado como medida de efetividade da atenção primária, partindo do pressuposto de que um desempenho mais satisfatório desse nível de atenção poderia resultar em diminuição do risco de internação para um conjunto de agravos que inclui hipertensão e diabetes, importantes fatores de risco para o IAM.

A disponibilidade de leitos no Grande ABC foi maior em 2000 e 2010do que na Baixada Santista, mas ainda assim insuficiente para atender às demandas da população. A falta de leitos nos hospitais tende a agravar a articulação entre o atendimento hospitalar e o pré-hospitalar, revelando importantes desigualdades no acesso à assistência.

Ainda durante esse período, embora a taxa de leitos de internação do Sistema Único de Saúde por 1.000 habitantes tenha apresentado redução em ambas as regiões, o total de leitos de unidade de terapia intensiva adulto ou coronariana por 100.000 habitantes aumentou, assim como os valores do indicador taxa de angioplastia padronizada por sexo e idade por 100.000 habitantes com 20 anos ou mais, que reflete a disponibilidade de serviços de atendimento cardiológico nas regiões.

Para acompanhar melhor esse cenário, foi analisado o comportamento anual da taxa de angioplastia padronizada por sexo e idade por 100.000 habitantes com 20 anos ou mais, considerando o mesmo período de estudo. Tanto no Grande ABC quanto na Baixada Santista houve um importante aumento na taxa de cirurgias cardíacas entre os anos de 2001 e 2003, período anterior à implantação do Samu na região de intervenção. A partir de 2004, ocorre redução no volume de angioplastia, padrão que se mantém até 2007, quando passa a ocorrer incremento no valor ao longo dos anos subsequentes. No entanto, na Baixada Santista, volta a ocorrer tendência decrescente a partir de 2009.

Considerando na análise outras intervenções que possam ter sido introduzidas no mesmo período de estudo, vale a pena destacar aqui a implantação de unidades de pronto atendimento (UPA), que têm sido consideradas importantes no diagnóstico de urgências na atenção às doenças cardiovasculares, em especial ao infarto agudo do miocárdio, ao se constituírem em unidades intermediárias entre a atenção primária e as emergências hospitalares. No entanto, apesar de formulada em 2008, a sua implantação em larga escala no país ocorreu entre 2011 e 2016, período não contemplado neste estudo^28^ .

Em relação à adoção da lei antifumo formulada em 2007, sua implementação de fato ocorreu no estado de São Paulo em 2009 com a Lei nº 13.541. Essa medida enfatizou a proibição do uso de cigarros e derivados em ambientes públicos e privados, exceto residências e locais de culto religioso e destinados ao consumo de produtos fumígenos, visando redução na mortalidade por doenças crônicas, com ênfase no câncer e na doença cardiovascular^29^ .

A maior força do estudo foi, além de usar o desenho quase experimental de séries temporais com grupo controle, controlar diferenças em ambos o nível e a tendência pré-existentes. No entanto, uma das limitações deste estudo é o baixo poder de generalização; portanto, suas conclusões só devem ser aplicadas a populações com um perfil semelhante. Resultados contraditórios podem ser parte das diferenças do contexto local. Além disso, é preciso considerar a conformação do Samu junto aos outros componentes da rede de urgência e emergência nos distintos territórios que compõem a região do Grande ABC. Nesse sentido, em estudos baseados em comunidade, o uso de desenho quase experimental irá frequentemente requisitar ambas as abordagens quantitativa e qualitativa para chegar a uma conclusão mais ampliada acerca dos efeitos da intervenção sob estudo^27 , 29 , 30^ .

Por fim, o resultado pouco expressivo (embora estatisticamente significativo) na tendência pós-intervenção da taxa de mortalidade hospitalar por IAM na região do Grande ABC pode levar a uma interpretação de que o efeito foi de baixo impacto. Entretanto, no caso de estudos ecológicos, os efeitos ocorrem igualmente na população como um todo. Assim, são esperados achados de efeitos menores, sem que isso signifique baixo efeito real sobre o fenômeno estudado. Além disso, a intervenção estudada pode ser uma condição que por si só não implica efetividade, mas sem a qual essa mesma efetividade não estaria presente, integrando um conjunto de condições que, agregadas, geram um resultado positivo.

## CONCLUSÃO

A complexidade da busca em estabelecer o efeito de uma dada intervenção reside no fato de que quase todos os fenômenos observados têm uma multiplicidade de causas. Nessa perspectiva, a proposta dos “experimentos quase naturais” por meio de condições traçadoras ou eventos marcadores tem sido uma prática recorrente, uma vez que é difícil, em muitas situações, isolar as causas e os efeitos dos fenômenos de interesse.

O presente estudo reforça a utilidade da abordagem quase experimental de séries interrompidas, tornando-se uma opção viável para análise de intervenção em políticas de saúde quando estudos randomizados não são factíveis. Os resultados sugerem que, embora a efetividade do Samu não possa ser considerada como adquirida, trata-se de uma estratégia cuja expansão precisa ser levada em conta na formulação e consolidação de políticas com foco nas urgências.
